# Cell Cycle-Dependent Transcription: The Cyclin Dependent Kinase Cdk1 Is a Direct Regulator of Basal Transcription Machineries

**DOI:** 10.3390/ijms23031293

**Published:** 2022-01-24

**Authors:** Jorrit M. Enserink, Pierre Chymkowitch

**Affiliations:** 1 Section for Biochemistry and Molecular Biology, Faculty of Mathematics and Natural Sciences, University of Oslo, 0316 Oslo, Norway; 2 Department of Molecular Cell Biology, Institute for Cancer Research, Oslo University Hospital, 0379 Oslo, Norway; 3 Centre for Cancer Cell Reprogramming, Institute of Clinical Medicine, Faculty of Medicine, University of Oslo, 0318 Oslo, Norway; 4 Department of Microbiology, Oslo University Hospital, 0372 Oslo, Norway

**Keywords:** cyclin-dependent kinase, Cdk1, RNA polymerase, transcription, cell cycle

## Abstract

The cyclin-dependent kinase Cdk1 is best known for its function as master regulator of the cell cycle. It phosphorylates several key proteins to control progression through the different phases of the cell cycle. However, studies conducted several decades ago with mammalian cells revealed that Cdk1 also directly regulates the basal transcription machinery, most notably RNA polymerase II. More recent studies in the budding yeast *Saccharomyces cerevisiae* have revisited this function of Cdk1 and also revealed that Cdk1 directly controls RNA polymerase III activity. These studies have also provided novel insight into the physiological relevance of this process. For instance, cell cycle-stage-dependent activity of these complexes may be important for meeting the increased demand for various proteins involved in housekeeping, metabolism, and protein synthesis. Recent work also indicates that direct regulation of the RNA polymerase II machinery promotes cell cycle entry. Here, we provide an overview of the regulation of basal transcription by Cdk1, and we hypothesize that the original function of the primordial cell-cycle CDK was to regulate RNAPII and that it later evolved into specialized kinases that govern various aspects of the transcription machinery and the cell cycle.

## 1. Introduction

The cyclin-dependent kinase Cdk1 is the master regulator of the cell cycle [1,2]. It was first discovered in genetic screens in the budding yeast *Saccharomyces cerevisiae* [3,4,5,6,7]. Due to historical reasons, Cdk1 is often also referred to as cdc2 in *Schizosaccharomyces pombe* and in vertebrate cells, and it is also known as Cdc28 in *S. cerevisiae*. It is a proline-directed kinase that phosphorylates a large number of proteins during the different stages of the cell cycle, thereby driving cell cycle progression and executing specific processes associated with the different cell cycle stages [1]. Cdk1 activity is intricately controlled by the cell, which is essential for the accurate transmission of genetic material from one generation to the next [8]. Failure to accurately control Cdk1 activity has been linked to genomic instability [9,10,11,12], and loss of cell cycle control lies at the heart of tumor growth [13].

**Figure 1:**
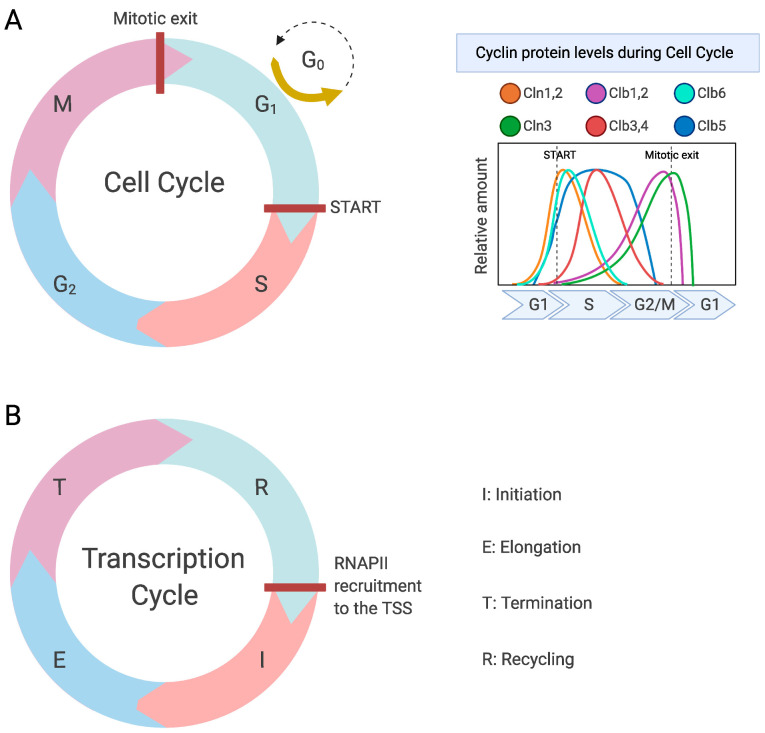
The cell cycle and the transcription cycle in *S. cerevisiae*. (**A**) The four phases of the cell cycle (**left**), which are regulated by Cdk1 in complex with nine different cyclins that fluctuate throughout the cell cycle (**right**). (**B**) The four main phases of the transcription cycle. Created with Biorender.com.

Regulation of the cell cycle by Cdk1 has been extensively reviewed elsewhere [1,2,14,15,16], and here we will only briefly recapitulate some of the key elements. Cdk1 is part of a family of CDKs that have been conserved in evolution. In budding yeast there exist six CDKs, of which only Cdk1 is essential for cell cycle regulation. Cdk1 associates with nine different cyclins to execute the various phases of the cell cycle (Figure 1A and Table 1). Another CDK, the non-essential CDK5-like kinase Pho85, which associates with ten different cyclins, supports efficient cell cycle progression and performs a heterogeneous set of functions that include nutrient signaling, stress responses, efficient budding, and DNA damage repair [17,18]. The remaining four CDKs associate with a single cyclin and are best known for regulating several aspects of transcription by RNA polymerase II (RNAPII; see below).

While Cdk1 is well known to regulate specific transcription factors to activate gene expression programs during the cell cycle (for reviews see [19,20,21,22,23]), several studies in vertebrate cells and in yeast have revealed that Cdk1 can also directly regulate the basal transcription machinery. The aim of this review is to provide an overview of these findings in the context of the evolutionary origins of these ancient processes.

## 2. Regulation of Transcription by RNA Polymerase II

### 2.1. Regulation of the Transcription Cycle

**Figure 2:**
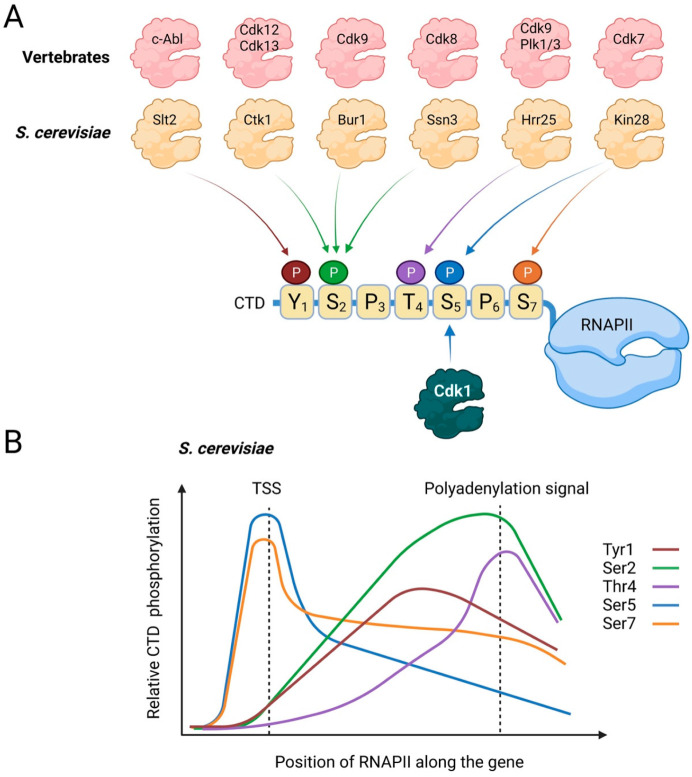
CTD phosphorylation. (**A**) Kinases that phosphorylate the CTD in vertebrates (**top**) and in *S. cerevisiae* (**bottom**). Additional CTD kinases have been described but are not depicted here for simplicity. For clarity, only a single heptad repeat is shown. (**B**) Relative levels of the different CTD residues along the body of genes in *S. cerevisiae*. Created with BioRender.com.

The most intensively studied RNA polymerase is RNAPII, which transcribes DNA into mRNA as well as most species of small nuclear RNA and microRNA. Synthesis of mRNA can be divided into four key steps that are often referred to as the transcription cycle (Figure 1B), i.e., initiation, elongation, termination, and recycling. These processes are in large part regulated through post-translational modifications (PTMs) of the C-terminal domain (CTD) of RNAPII. In budding yeast, the CTD contains 26 repeats of the heptapeptide consensus sequence Tyr_1_-Ser_2_-Pro_3_-Thr_4_-Ser_5_-Pro_6_-Ser_7_, whereas the mammalian RNAPII-CTD contains 26 similar repeats followed by 26 repeats of more diverging sequences [24]. Many PTMs of the CTD have been described, including ubiquitination, sumoylation, O-GlcNAcylation, isomerization of proline residues, methylation of arginine and lysine residues in non-consensus repeats, lysine acetylation, and arginine citrullination [25,26]. However, by far the best-characterized modification of the CTD is phosphorylation. As we will discuss below, the hypophosphorylated form of RNAPII is considered to be the inactive form that is recruited to the PIC [27], whereas dynamic phosphorylation of different CTD residues is associated with specific aspects of the transcription cycle. During the transcription cycle, several kinases are known to target these residues, primarily CDKs but also other kinases (Figure 2A), and the phosphorylation levels of these different residues rise and fall during the transcription cycle (Figure 2B). The compendium of CTD modifications is often referred to as the CTD code, which can be read by certain proteins to execute specific functions.

### 2.2. Transcription Initiation

**Figure 3:**
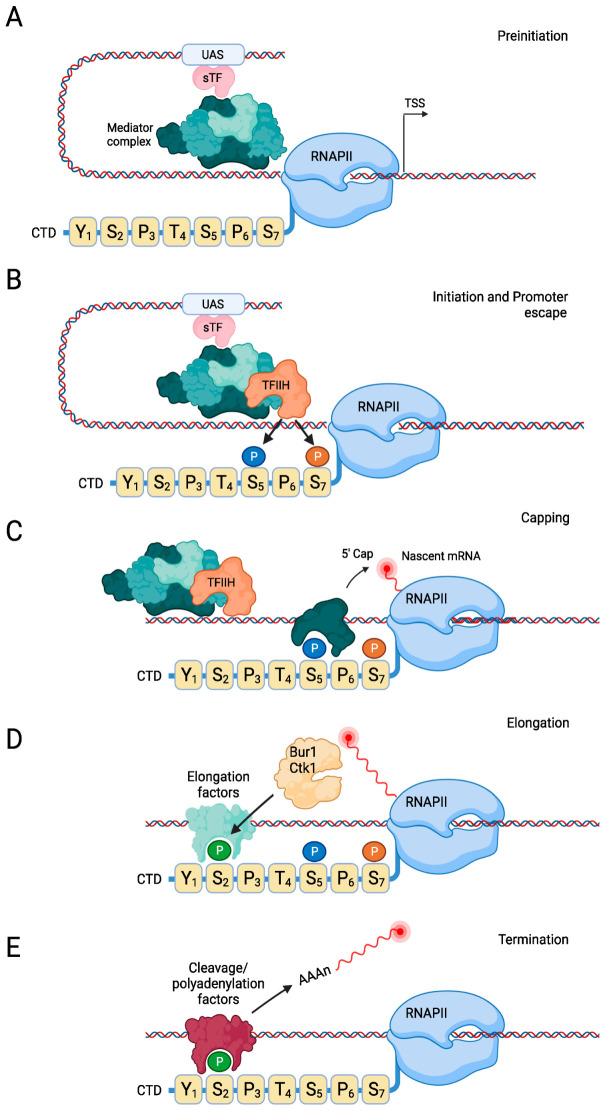
Stages of transcription by RNAPII. (**A**) During the preinitiation stage, upstream activation sequences (UAS) recruit specific transcription factors (‘sTF’), which bind the Mediator complex. Mediator recruits hypophosphorylated RNAPII to form the PIC. (**B**,**C**) Kin28 phosphorylates Ser5 and Ser7 when RNAPII initiates transcription, resulting in recruitment of the capping machinery and capping of the nascent mRNA. (**D**) During the elongation stage, Bur1 and Ctk1 phosphorylate Ser2, leading to binding of elongation factors. (**E**) When the polymerase reaches the polyadenylation signal in the gene, polyadenylation and cleavage factors that recognize phosphorylated Ser2 then cleave and polyadenylate the mRNA, followed by dissociation of the RNAPII complex. For clarity, only a single heptad repeat is shown in this figure. Created with Biorender.com.

Transcription begins with the initiation phase, which typically first involves binding of specific transcription factors (sTFs) to upstream activating sequences (Figure 3A). These sTFs often respond to environmental cues and the state of the cell, allowing the cell to respond to environmental changes or to regulate cell cycle progression. Hypophosphorylated RNAPII and six general transcription factors (gTFs), TFIIA, B, D, E, F, and H, are then recruited to the TSS to form the preinitiation complex of transcription (PIC). Assembly of the PIC can take place via either TFIID or SAGA [28,29,30], although in *S. cerevisiae* the vast majority of promoters are TFIID dependent [31,32]. TFIID makes contact with the sTFs bound to upstream activating sequences (UAS) and recruits the TATA binding protein (TBP; a component of TFIID) to the promoter [33]. Several other gTFs are then recruited to complete formation of the PIC, such as TFIIB, which makes contact with TBP, DNA, and RNAPII, as well as other gTFs that help stabilize the PIC on the chromatin. Transcriptional co-activators, such as the Mediator complex, are important transcriptional regulators that control PIC assembly and RNAPII activity at these early stages of transcription [34]. The yeast Mediator complex consists of 25 subunits and serves as a bridge between UAS-bound activators and the gTFs at the core promoter by forming chromatin loops. A notable component of the Mediator complex is a kinase module containing Ssn3 (Cdk8 and Cdk19 in mammals) [35,36,37]. This kinase module is generally associated with transcriptional repression, and it is released from the promoter during transcriptional activation. Dissociation of the kinase module is necessary for the interaction between Mediator and the PIC, but exactly how the kinase module interferes with PIC assembly remains to be determined—although one inhibitory function of this module involves phosphorylation of the CTD of RNAPII, since phosphorylated RNAPII is not recruited to the PIC [38]. The kinase module also inhibits the positive effects of the Mediator complex by phosphorylating the Mediator subunit Med2 [39]. Upon release of the Mediator kinase module, the PIC transitions into the so-called open complex, during which TFIIH separates the DNA strands at the transcription start site [40,41,42]. Mediator then stimulates the kinase activity of the TFIIH subunit Kin28 (Cdk7 in mammals) [43], which phosphorylates Ser5 of the CTD, as well as Ser7 (Figure 3B) [44]. CTD-Ser5 phosphorylation appears to be important for removing the Mediator complex from the promoter to allow RNAPII to escape the promoter [45]. Mediator and CTD-Ser5 phosphorylation may also regulate transcription initiation via liquid–liquid phase separation [46,47], which is important for transcriptional initiation and transition to the elongation phase.

### 2.3. Transcription Elongation

The elongation phase marks the productive phase of RNAPII. During this stage, several mRNA processing factors are recruited that mediate splicing and modification of the mRNA molecule. One such factor is the mRNA capping complex, which is recruited to the CTD upon TFIIH-mediated phosphorylation of CTD-Ser5 (Figure 3C). In addition, several chromatin-modifying enzymes associate with the polymerase to co-transcriptionally modify the chromatin, such as methylation and acetylation of histones. As the polymerase progresses, CTD-Ser5 gradually becomes dephosphorylated due to the action of the phosphatase Ssu72 [48], while phosphorylation of CTD-Ser2 increases due to the activity of the kinases Bur1 and Ctk1 (Figure 3D), which are the yeast counterparts of mammalian Cdk9 and Cdk12/13, respectively. Ctk1 is responsible for the bulk of CTD-Ser2 phosphorylation and acts primarily at the 3′ end of the gene, whereas Bur1 may act more towards the 5′ end of the gene [49]. Recruitment of Bur1 is mediated by its binding to phosphorylated CTD-Ser5, whereas Ctk1 is recruited by Bur1-mediated CTD-Ser2 phosphorylation [49]. Bur1 also phosphorylates the elongation factor Spt5 (DSIF in mammals), which promotes recruitment of the polymerase-associated factor (PAF) complex [50]. The PAF complex has multiple functions in transcription, including epigenetic modifications, RNA processing, and RNAPII recycling [51,52]. 

Stable association of Ctk1 with the CTD also involves binding of Spt6 to phosphorylated CTD-Ser2, which may set up an Sp6-Ctk1 feedforward loop [53]; Spt6 is an elongation factor that also serves as a histone chaperone that mediates nucleosome reassembly in the wake of RNAPII [54,55], and which promotes H3K36 methylation by the Set2 histone methyltransferase [56]. In addition to CTD-Ser2 phosphorylation, recruitment of Spt6 is also stimulated by phosphorylation of CTD-Tyr1 [57].

Phosphorylation of Tyr1, Thr4, and Ser7 is not as well understood as Ser2 and Ser5 phosphorylation [58]. In budding yeast, CTD-Tyr1 can be phosphorylated by the MAPK Slt2, which is particularly important for cellular homeostasis under certain stress conditions [59]. Importantly, phosphorylated CTD-Tyr1 has been shown to recruit the elongation factor Spt6, thereby blocking the recruitment of transcription termination factors and preventing premature termination of transcription [59]. Phosphorylation of Ser7 is mainly mediated by Kin28 and to a lesser extent by Bur1, and may be important for the recruitment of the capping machinery in *S. pombe* [60] and possibly also in *S. cerevisiae*, whereas in human cells it is involved in the expression and processing of snRNA [61,62]. Finally, Thr4 can be phosphorylated by several kinases in *S. cerevisiae*, although the most active Thr4 kinase appears to be Hrr25 [63]. Phosphorylation of Thr4 is not essential in *S. cerevisiae*, and it has been associated with chromatin remodeling, efficient mRNA splicing, and with the recruitment of the transcription termination machinery [63,64,65,66].

### 2.4. Transcription Termination

As the elongating polymerase progresses, it recruits transcription termination factors that terminate transcription. Termination and processing regions in the 3′ untranslated region of the nascent mRNA are recognized by the cleavage and polyadenylation factor (CPF)–cleavage factor (CF) complex [67]. This complex also binds to Ser2-phosphorylated repeats in the CTD, which is highest at the 3′ end of the gene (Figure 3E) [67]. The RNA is then cleaved by the endonuclease subunit of the CPF complex at the poly(A) site [68]. Interestingly, the CPF complex also contains a subunit with phosphatase activity, Glc7, which dephosphorylates CTD-Tyr1 at the polyadenylation site, resulting in recruitment of the termination factors Pcf11 and Rtt103 [69]. After RNA cleavage, the polymerase complex briefly continues synthesizing RNA, after which it is released from the DNA. Exactly how the polymerase terminates is not entirely clear, and two models exist that are not mutually exclusive: The torpedo model and the allosteric model [67,70,71,72]. In the torpedo model, a 5′-3′ exonuclease is recruited to the RNA that keeps protruding from the polymerase after cleavage of the precursor mRNA by the CPF–CF complex. As the nuclease quickly degrades the RNA, it may catch up with the polymerase to induce its dissociation from the DNA. In the allosteric model, binding of the CPF–CF complex induces an allosteric change to the elongating polymerase, causing the release of elongation factors. The polymerase then loses processivity and eventually terminates and dissociates.

### 2.5. RNAPII Recycling

Finally, highly transcribed genes are thought to recycle the polymerase to sustain high transcription rates, which has been best studied in the context of short, highly transcribed RNAs by RNAPIII, such as tRNAs (see below). Previous studies have indicated a role for gene looping in the recycling of RNAPII, which depends on interactions between transcription initiation and termination machineries, such as the TFIIB and CPF complexes [73,74,75]. For instance, the CTD-S5 phosphatase Ssu72 interacts with the CPF complex as well as with TFIIB [76,77], and this interaction has been suggested to form a gene loop to promote the transfer of RNAPII from the terminator to the promoter [78]. More recent studies have shown that RNAPII recycling requires the PAF complex member PAF1 in human cells [79], but exactly how PAF1 mediates this effect remains unclear. Compared to the recycling of RNAPIII (see below), much still remains to be learned about this process. For efficient incorporation of RNAPII into the PIC to occur, it has to be in a hypophosphorylated form [27]. Several phosphatases have been described that can dephosphorylate the CTD [80]. In addition to the CTD-Ser5 phosphatase Ssu72 mentioned above, the CTD-Ser2 phosphatase Fcp1 has been linked to RNAPII recycling [81,82,83]. Other CTD phosphatases include Rtr1 and Cdc14; however, their function in recycling remains to be firmly established [80].

## 3. Regulation of RNAPII by Cdk1 during the Cell Cycle

Both in mammalian and in yeast cells, cell-cycle CDKs regulate specific transcription factors to activate gene expression programs that control and execute the different stages of the cell cycle. This has been extensively reviewed elsewhere and will not be repeated here (see [1,19]). Instead, we will focus on the direct regulation of the basal transcription machinery by Cdk1.

### 3.1. Downregulation of the Basal Transcription Machinery during M Phase in Vertebrate Cells

**Figure 4:**
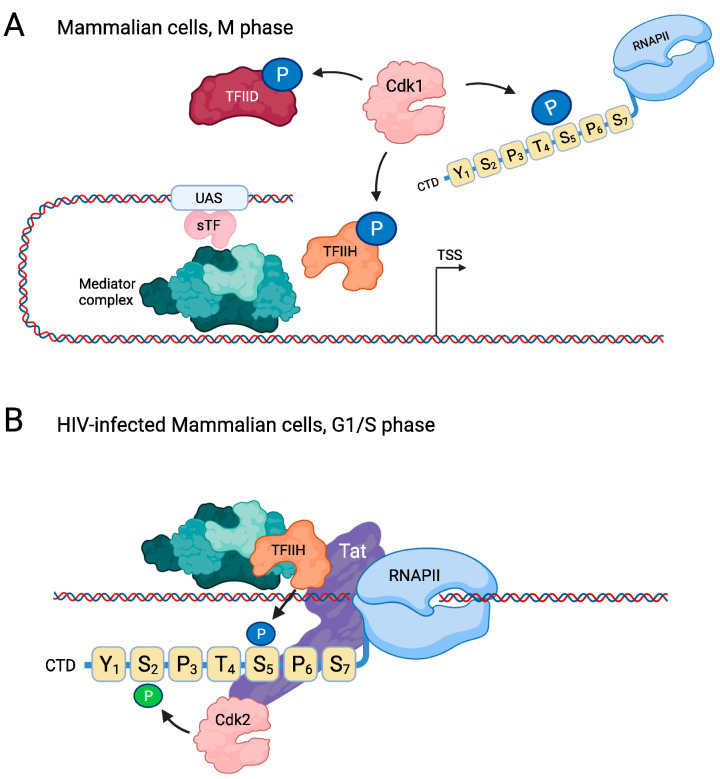
Regulation of RNAPII by cell/cycle CDKs in mammalian cells. (**A**) In M phase, Cdk1 inhibits transcription by phosphorylating RNAPII on Ser5, thereby preventing its incorporation into the PIC. Cdk1 also phosphorylates and thereby inhibits the Cdk7 subunit of TFIIH as well as multiple components of TFIID. (**B**) In contrast, during G1/S phase the cell-cycle CDK Cdk2 activates transcription in HIV/infected cells. Here, the Tat protein recruits Cdk7, which enhances Ser5 phosphorylation, but Tat also recruits Cdk2, which subsequently phosphorylates Ser2 to promote transcriptional elongation. For clarity, only a single heptad repeat is shown in this figure. Created with Biorender.com.

Although minimal levels of transcription are maintained during mitosis, especially at housekeeping genes, transcription is globally reduced during M phase when chromatin becomes highly compacted [84,85,86,87]. In addition to the overall inability of the polymerase to access most genes [88], several gTFs are inactivated by direct mitotic phosphorylation (Figure 4A). For instance, in mammalian cells, multiple TFIID subunits are phosphorylated by Cdk1 during M phase, including TBP and the TBP-associated factors TAFn20/15, TAFn31, and TAFu80, and this mitotically phosphorylated form of TFIID is incapable of initiating transcription [89]. Furthermore, mitotic phosphorylation by Cdk1 of Ser164 in the T loop of the TFIIH subunit Cdk7 inhibits its catalytic activity to repress transcription in M phase [90,91]. Cyclin B-Cdk1 has also been shown to phosphorylate the CTD of RNAPII in M phase, resulting in transcriptional inhibition [92]. More specifically, Cdk1 has been reported to phosphorylate Ser2 and Ser5 [92,93,94,95], and one potential mechanism for transcriptional inhibition is that the phosphorylated form of RNAPII cannot be recruited to the PIC [92,96].

In addition to this negative effect of Cdk1 on RNAPII-mediated transcription during M phase, there are indications that cell-cycle CDKs positively regulate RNAPII during interphase, such as during a human immunodeficiency virus-1 (HIV-1) infection. Expression of the HIV-1 genome requires the assembly of the host cell’s basal transcription factors on the HIV-1 promoter, which is located in the 5′ long terminal repeat of the viral genome [97]. HIV-1 expression requires the viral transactivator protein (Tat), which mediates transcriptional elongation of HIV-1 via hyperphosphorylation of the RNAPII CTD (Figure 4B). Tat stimulates several CTD kinases, including Cdk7 and Cdk9. However, Tat also binds and recruits Cdk2, which then preferentially phosphorylates the CTD on Ser2 to enhance HIV-1 transcription [98,99]. This may explain the cell cycle-dependent expression of HIV-1, which preferentially occurs at the G1–S boundary [100]. Whether cell-cycle CDKs are also important for positive regulation of transcription of non-viral genes is not clear.

### 3.2. Activation of the Basal Transcription Machinery by Cdk1 in Budding Yeast

**Figure 5:**
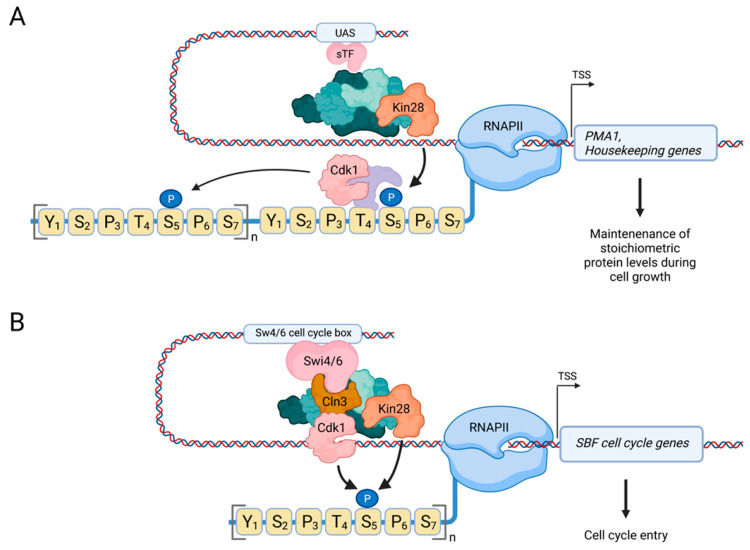
Cdk1 directly controls RNAPII by phosphorylating CTD-Ser5 in budding yeast. (**A**) Regulation of RNAPII by Cdk1 at housekeeping genes. Cdk1 localizes to highly expressed genes, such as *PMA1*, where it phosphorylates Ser5 to promote transcription and recruitment of the capping machinery [103]. Cdk1 synergizes with Kin28 in this process [103], and we have previously hypothesized that priming of RNAPII by Kin28 results in recruitment of Cdk1, potentially through binding of its cyclin partner to phosphorylated Ser5, which then phosphorylates additional Ser5 residues in the CTD [109]. (**B**) Regulation of RNAPII by Cdk1 at cell cycle genes. During late G1, Cln3-Cdk1 is recruited to SBF target genes where it directly promotes transcription by phosphorylating CTD-Ser5 to promote cell cycle entry. Created with Biorender.com.

In contrast to vertebrate cells, budding yeast cells do not appear to undergo an equally dramatic global reduction in transcriptional activity during M phase [101,102]. There is also no evidence for phosphorylation of Spt15 (yeast TBP) by Cdk1, or any of the other components of TFIID. Furthermore, Ser164 in Cdk7, which is negatively regulated by Cdk1 in human cells, is not conserved in Kin28. However, there is evidence for direct regulation of RNAPII by Cdk1 to positively control transcription. For instance, using ChIP-seq, we have shown that Cdk1 localizes to a number of highly expressed genes [103], including *PMA1*, which encodes a proton pump and which is often used as a transcriptional model gene due to its high expression levels [104,105]. *PMA1* encodes a long-lived protein that is asymmetrically distributed to the mother cell after each cell division, whereas the daughter cell is born with very low levels of Pma1 [106,107]. Given that Pma1 is essential for cellular homeostasis throughout the cell cycle, there is a clear need for the daughter cell to quickly synthesize new Pma1. Indeed, *PMA1* has long been known to be expressed in a cell cycle-dependent manner [108], even though its promoter lacks binding sites for classic cell cycle-regulated transcription factors. So how is cell cycle-dependent transcription of *PMA1* regulated? We have shown that Cdk1 can promote RNAPII activity at *PMA1* by directly phosphorylating CTD-Ser5, which is also important for efficient capping of *PMA1* mRNA, and that Cdk1 cooperates with the TFIIH kinase subunit Kin28 in this process [103] (Figure 5A). 

Interestingly, the recruitment of Cdk1 and Kin28 is mutually dependent and requires the catalytic activity of both kinases, suggesting the existence of a positive feedback mechanism that reinforces the transcriptional activity of RNAPII at *PMA1*. While the exact molecular mechanism remains to be determined, we have previously proposed a model in which Cyclin–Cdk1 complexes bind phosphorylated CTD-Ser5 via a priming mechanism by Kin28 [109] (Figure 5A); this model is inspired by the earlier, unrelated finding that Clb5-Cdk1 is recruited to its target Sic1 via an initial priming phosphorylation on Sic1 by an earlier acting Cyclin–Cdk1 complex [110]. Notably, a priming model has also been proposed for CTD phosphorylation by Cdk2 in mammalian cells [98]. A cooperative function of Cdk1 and Kin28 in regulating the basal transcription machinery is also in accordance with the previously described requirement of Cdk1 kinase activity in the recruitment of TFIIH, of which Kin28 is a subunit [111].

It remains unclear why Cdk1 has a positive function in regulation of the basal transcription machinery in budding yeast, whereas in vertebrate cells it appears to have the opposite function. One explanation could be that vertebrate studies have mainly focused on transcriptional inhibition that occurs during M phase, which is mediated by Cyclin B–Cdk1 complexes, and that a positive function in the regulation of the basal transcription machinery by Cdk1 or other cell-cycle CDKs that act earlier in the cell cycle (such as Cdk2, Cdk4, or Cdk6) has simply been overlooked. Guided by the recent results from budding yeast, new vertebrate studies should provide deeper insight into this process.

### 3.3. Direct Regulation of RNAPII by Cdk1 Promotes Cell Cycle Entry

Building on our earlier findings that Cdk1 is a CTD kinase, it was recently shown that Cdk1 also directly phosphorylates RNAPII CTD-Ser5 at cell cycle-regulated genes [112] (Figure 5B). Here, Cdk1 is recruited to cell cycle-regulated genes by the Swi4/6-dependent cell-cycle box-binding factor (SBF) transcription factor complex. SBF has a well-known function in the expression of an early cell cycle program that includes the cyclin genes *CLN1,2* and *CLB5,6* to induce cell cycle entry [19]. It was previously demonstrated that Cln3-Cdk1 phosphorylates Whi5 in late G1, leading to dissociation of Whi5 from SBF and activation of transcription, in a manner not unlike regulation of Rb by Cdk1 in vertebrates [113,114]. However, a new study shows that Cln3-Cdk1 also directly phosphorylates RNAPII CTD-Ser5 to stimulate the transcription machinery at SBF target genes to promote cell cycle entry [112]. Therefore, this recent study confirms and extends our earlier findings that Cdk1 is a transcriptional CDK that phosphorylates Ser5.

It will be interesting to determine whether a similar system also operates in mammalian cells. Given that early cell cycle events are mainly executed by Cdk4/6, in-depth mammalian studies may also have relevance for human disease, because Cdk4/6 inhibitors have gained attraction in the treatment of various forms of cancer, particularly breast cancer [115]. Cdk4/6 promote cell cycle entry by phosphorylating Rb, which is an inhibitor of the transcription factor E2F; phosphorylation of Rb results in its release from E2F, leading to E2F activation [116]. E2F serves as an activator of a transcriptional program that includes cyclin genes, thereby setting up a potent positive feedback loop in which rapidly increasing cyclin-CDK activity leads to further inhibition of Rb, thus stimulating cell cycle entry in a switch-like manner. Although Cdk4/6 inhibitors have a multitude of effects on cancer cells, one important effect is believed to be the breaking of this feedback loop by preventing phosphorylation of Rb and its dissociation from E2F, such that E2F cannot activate transcription and the cells arrest at the G1/S boundary [117]. If, in addition to Rb, Cdk4/6 also directly control RNAPII activity during cell cycle entry via CTD phosphorylation, then the molecular mechanism by which Cdk4/6 inhibitors exert their effect in the treatment of cancer may need to be revisited.

### 3.4. Indirect Regulation of Basal Transcription by Cdk1

In addition to phosphorylating the CTD, Cdk1 can also regulate the activity of the RNAPII machinery more indirectly. For instance, Cdk1 has been reported to phosphorylate the yeast nuclear pore complex protein Nup1, which promotes the localization of highly transcribed genes to the nuclear pore, including *GAL1* [118]. The nuclear pore is known to constitute a nuclear subcompartment where high transcription rates take place, and where transcription is coordinated with efficient RNA processing and export [119,120,121]. 

It is also important to mention that Cdk1 has a kinase-independent function in the regulation of transcription by RNAPII. In this process, Cdk1 and its interaction partner Cks1 are recruited to the promoter regions of cell cycle genes, such as *CDC20*, as well as non-cell cycle genes, such as *GAL1*, to promote the recruitment of the proteasome, which is important for transcriptional regulation through nucleosome eviction [122,123,124].

Taken together, Cdk1 has multiple kinase-dependent and -independent functions in the regulation of the basal transcription machinery in budding yeast, which involves phosphorylation of CTD-Ser5, recruitment of the proteasome, as well as localization of genes to a nuclear subcompartment conducive for high transcription rates. Direct regulation of RNAPII by Cdk1 is not only important for cell cycle progression, but also for maintenance of protein stoichiometry and cellular homeostasis by promoting the expression of housekeeping genes during the cell cycle.

### 3.5. Is Phosphorylation of RNAPII-CTD the Original Function of Cdk1?

Interestingly, CDKs and the CTD of RNAPII have been suggested to have co-evolved during evolution [125]. Comprehensive analysis of the CTD revealed that it probably originated as tandemly repeated heptads before the divergence of extant eukaryotic taxa, and that those taxa without recognizable CTD repeats have undergone degeneration [126]. The CTD may have evolved from YSP and SP sequences at the C-terminal end of the catalytic subunit of RNAPII, resulting in the formation of a YSPxSPx motif [24,126]. This motif likely provided a regulatory opportunity with a substantial fitness advantage, which may have been an early essential function that also evolved in the common ancestor of extant eukaryotes, such as cotranscriptional pre-mRNA splicing or mRNA capping [126]. The initial YSPxSPx motif was rapidly duplicated into tandemly repeated motifs that are found in one form or another in all eukaryotes [126]. A few organisms have apparently lost a large portion of the tandem CTD repeats, such as the trichomonad *Pentatrichomonas* which only has a single YSPASPL sequence (although it has many SP sites in the C-terminus of RNAPII) [126].

Of note, Ser5 in the YSPxSPx motif is conserved in all eukaryotes, and proline-directed phosphorylation is a key feature of CDKs. Indeed, it has been argued that CDKs co-evolved with the CTD of RNAPII [125,127], with the formation of the CTD as a potential driving force for CDK diversification. This may have provided organisms with regulatory opportunities to respond to changes in their environment and to provide opportunities to quickly adapt to a new niche. Given that (i) the classical transcriptional CDKs arose after the cell-cycle CDKs [125,128]; (ii) that oscillating transcription is key to cell cycle control [19]; and (iii) that cell-cycle CDKs are direct regulators of the CTD [92,103,109], we hypothesize that the original function of the archetypical cell-cycle CDK was first and foremost to directly control RNAPII, and that only later in evolution the cell-cycle CDKs branched out to regulate additional aspects of the cell cycle.

## 4. Regulation of Transcription by RNA Polymerase I

RNAPI is responsible for the transcription of all ribosomal RNA except 5S RNA, which is synthesized by RNAPIII. RNAPI accounts for approximately 60% of transcriptional activity in eukaryotes [129]. In budding yeast, rDNA is organized in ~150–200 rDNA repeats in a single locus, although most of these repeats are kept in a transcriptionally inactive state [130]. RNAPI transcribes a single 7 kb pre-rRNA, which is processed to yield mature rRNA.

### 4.1. The RNAPI Transcription Cycle

**Figure 6:**
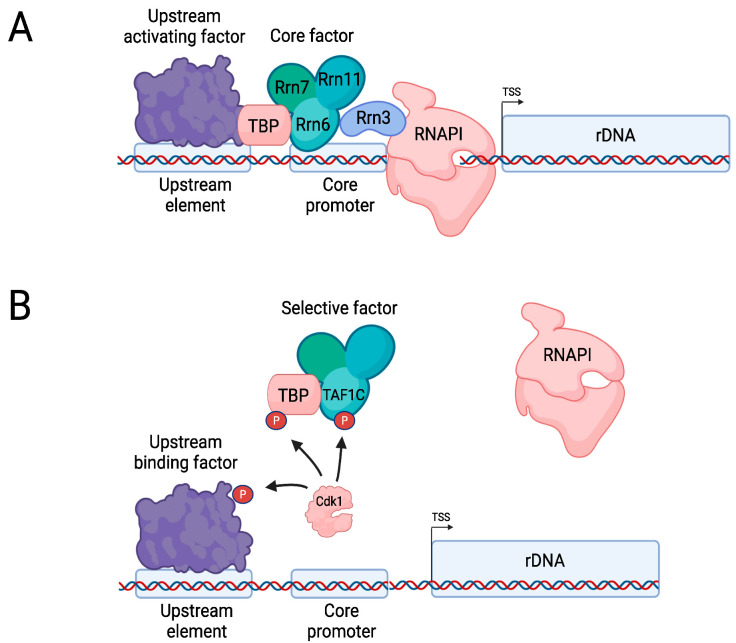
Regulation of RNAPI. (**A**) Overview of rDNA gene transcription by RNAPI (budding yeast nomenclature). (**B**) In mammalian cells, during M phase Cyclin B-Cdk1 phosphorylates TAF1C, TBP, and upstream binding factor, which prevents binding of Selective factor to the promoter. Dephosphorylation by the phosphatase Cdc14 allows for reactivation of transcription (not depicted here). Created with Biorender.com.

RNAPI transcription begins with initiation (Figure 6A), which involves the initiation factors Rrn3; Core Factor, which consists of Rrn6, Rrn7, and Rrn11; TBP; and upstream activating factor (AUF), which consists of histones H3 and H4, Uaf30, Rrn5, Rrn9, and Rrn10 [131]. AUF binds an upstream element in the rDNA promoter and makes contact with Core Factor, which is located at the core promoter. Rrn3 binds to RNAPI and this makes the polymerase competent for transcription initiation. Core Factor promotes binding of the Rrn3–RNAPI complex to DNA and positions it correctly at the TSS, whereas the interaction of TBP with Core Factor further enhances the transcription rate of RNAPI [131]. Compared to RNAPII, transcriptional elongation by RNAPI is relatively efficient in the absence of extrinsic factors elongation due to intrinsic elongation-promoting subunits, such as A34 and A49 [132]. Several trans-acting factors have been identified that promote RNAPI elongation rates, such as the PAF complex and Spt4/Spt5 [133,134]. Processing of the pre-rRNA occurs co-transcriptionally and the rate of elongation effect is known to affect this process [135,136]. Termination of RNAPI requires a stretch of DNA at the 5′ end of the rDNA repeat containing a 10–15 bp-long T-rich stretch as well as a downstream Reb1 binding site, which is bound by the Reb1 homolog Nsi1 [137,138]. The polymerase may stall at these DNA elements, destabilizing it and causing dissociation from the template [138]. Other factors involved in termination include rRNA processing factors such as the endonuclease Rdn1, the exonuclease Rat1, the helicase Sen1, and the chromatin-modifying factors Chd1, Isw1, and Isw2 [139].

### 4.2. Regulation of RNAPI during the Cell Cycle

The activity of RNAPI has been shown to be dependent on environmental conditions, particularly nutrient status [133,140]. Although more than 100 phosphorylation sites have been identified along the 14 RNAPI subunits [141], the physiological significance of the vast majority of phosphorylation sites remains unknown, and only very few kinases have been identified (for a review see [141]). Nonetheless, it is well known that the cell carefully controls rDNA transcription during the cell cycle, and CDKs downregulate rDNA transcription in M phase both in vertebrate cells and in yeast.

In vertebrate cells, Cyclin B-Cdk1 phosphorylates Selective Factor 1 [142], which is the TBP-containing mammalian counterpart of Core Factor that further consists of TAF1C (Rrn6), TAF1B (Rrn7), and TAF1A (Rrn11) as well as the additional subunits TAF1D and TAF12 [143,144] (Figure 6B). Although Cdk1 phosphorylates both TBP and TAF1C, it appears that phosphorylation of TAF1C is particularly important, abrogating the interaction with upstream binding factor (UBF; the mammalian counterpart of UAF) and thereby inactivating transcription of rDNA [142,145,146]. UBF itself is also a target of inhibitory mitotic phosphorylation by Cdk1 [147]. Dephosphorylation of TAF1C by Cdc14 allows for reactivation of rDNA transcription when cells exit from mitosis [146].

In budding yeast, rDNA transcription is also inhibited in M phase [148], although the mechanism appears to be very different from mammalian cells. More specifically, rDNA is silenced during mitosis to allow for the loading of the Condensin complex, and this is important for accurate chromosome separation [148]. In contrast to mammalian cells, where the phosphatase Cdc14 reactivates RNAPI after exiting from mitosis [146], in budding yeast the phosphatase activity of Cdc14 results in rDNA silencing during anaphase [148]. This appears to have multiple effects on RNAPI, including nucleolar exclusion of some but not all RNAPI subunits and the loss of RNAPI binding to chromatin [148]. Although the critical substrates in this process remain to be identified, one potential Cdc14 substrate may be the RNAPI subunit Rpa43 [148]. However, the Rpa43 kinase remains unknown and is unlikely to be Cdk1 since the reported phosphorylation sites in Rpa43 are all non-proline directed (https://thebiogrid.org/34723/protein, accessed on 19 December 2021). In conclusion, whereas Cdk1 has a clear function in direct regulation of RNAPI in mammalian cells, whether this function is conserved in budding yeast remains unclear.

## 5. RNA Polymerase III

### 5.1. The RNAPIII Transcription Cycle

**Figure 7:**
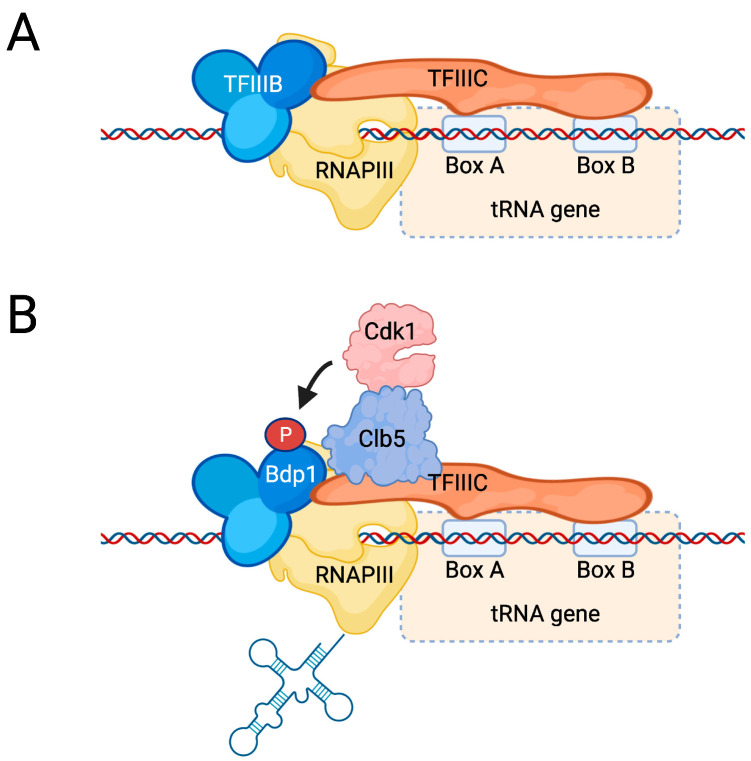
Regulation of RNAPIII. (**A**) tRNA genes have an internal promoter that consists of an A box and a B box, which is bound by TFIIIC. TFIIIC interacts with TFIIIB, leading to recruitment of RNAPIII and transcription. (**B**) Cdk1 is recruited to tRNA genes by the cyclin Clb5, although it is unknown which protein is recognized by Clb5. Cdk1 then phosphorylates Bdp1, which promotes its interaction with TFIIIC, resulting in increased tRNA synthesis.

RNAPIII synthesizes non-coding RNAs such as tRNA, 5S RNA, and long non-coding RNA (lncRNA). RNAPIII consists of 17 subunits and functions together with three transcription factor complexes, i.e., TFIIIA, TFIIIB, and TFIIIC [149]. TFIIIA is a single protein called Pzf1/Tsc2 in budding yeast [150] and is required only for transcription of *RDN5*, which encodes 5S rRNA. TFIIIB consists of three subunits, Brf1, Bdp1, and TBP, whereas TFIIIC is a large complex consisting of six subunits. Here, we will mainly focus on the regulation of transcription of tRNA genes, which are best studied. There are 275 tRNA genes in budding yeast, which have an internal promoter that consists of two elements, termed the A box and B box (Figure 7A). These elements are recognized by TFIIIC, which recruits TFIIIB upstream of the TSS. TFIIIB then recruits RNAPIII, which involves contacts between the TFIIIB subunit Brf1 and the RNAPIII subunit Rpc34 within the pre-initiation complex [151], resulting in the melting of double-stranded DNA at the promoter [152,153]. Brf1 also interacts with the Rpc82 subunit of RNAPIII, and mutations that abrogate this interaction result in slow growth at non-optimal growth temperatures [154]. During elongation, interactions between Brf1 and the polymerase may be displaced by the nascent RNA [155]. Termination of RNAPIII is signaled by a tract of A residues on the template DNA strand, typically 5–8A in length in budding yeast [156], and termination involves weak base-pairing interactions between the template’s oligo(dA) strand and the oligo(U) in the nascent RNA that acts as a destabilizing signal [157]. RNAPIII is well known to rapidly reinitiate transcription in a process called facilitated recycling, which requires TFIIIB [158], and it has been reported that TFIIIB stays attached to RNAPIII throughout the transcription cycle [155].

### 5.2. Regulation of RNAPIII by Environmental Cues

Given that the RNA molecules synthesized by RNAPIII account for ~20% of total cellular RNA and that their synthesis consumes up to ~15% of nucleotides used in transcription [159], it is not surprising that RNAPIII activity is strongly dependent on environmental signals, including nutrient status, DNA damage, and heat stress [159,160]. In budding yeast, a major regulator of RNAPIII is Maf1 [161]. Maf1 cycles between a phosphorylated and unphosphorylated state [162]. Under optimal growth conditions, Maf1 exists as a phosphoprotein that is exported from the nucleus by Msn5 [163]. Kinases that phosphorylate Maf1 include TORC1, the Tor-dependent kinase Sch9, PKA, and CK2 [164,165,166,167]. However, during environmental stress, such as nitrogen deprivation, which results in the inactivation of TORC1, Maf1 is dephosphorylated by PP4 and PP2A, allowing it to enter the nucleus [162,168,169]. Unphosphorylated Maf1 then interacts with RNAPIII and prevents it from reinitiating transcription [170].

The cell also regulates RNAPIII directly. For instance, when nutrient levels are suboptimal for growth and TORC1 is inactivated, expression of the kinase Kns1 increases and it enters the nucleus to phosphorylate the Rpc53 subunit of RNAPIII on T232 [171]. This serves as a priming site for further phosphorylation by the GSK3 kinase on S224 and T228, resulting in reduced RNAPIII activity in a manner dependent upon Maf1 [171]. It is not exactly clear how these phosphorylations impair the activity of RNAPIII, but it has been speculated that they could interfere with facilitated recycling or promote dissociation of the polymerase from the template to allow binding of Maf1 [171]. In addition to phosphorylation, we have found that RNAPIII is regulated by sumoylation [172,173], which promotes RNAPIII activity [160,173]. It is not entirely clear how Sumo enhances RNAPIII activity, but it could be through stabilization of the holoenzyme [173]. Sumoylation of RNAPIII is highly sensitive to environmental conditions, and environmental insults cause desumoylation of RNAPIII subunits and loss of transcriptional activity [160].

### 5.3. Regulation of RNAPIII Activity by Cdk1 

tRNAs were long thought to be extremely stable, with half-lives up to several days [174]. However, recent studies have revealed that tRNA stability is in fact highly regulated and subject to rapid turnover, particularly during cell stress such as nutrient starvation [175,176]. Accordingly, tRNA synthesis has been found to fluctuate during the cell cycle [177,178,179,180]. In mammalian cells, Cyclin B-Cdk1 inactivates tRNA transcription during mitosis by phosphorylating TFIIIB [85,181,182]. Cdk1 phosphorylates several substrates in TFIIIB, including TBP, although TBP does not appear to be the critical target for inactivation of tRNA transcription [182]. Instead, phosphorylation of Brf1 results in the release of Bdp1 from chromatin, whereas Brf1 and TBP remain associated [181].

In contrast to mammalian cells, Cdk1 appears to have a positive effect on tRNA transcription in budding yeast [179,180]. For instance, we recently demonstrated that Cdk1 promotes cell cycle-dependent tRNA synthesis by directly regulating the RNAPIII machinery [180] (Figure 7B). In this process, Cdk1 is recruited to tRNA genes via its cyclin Clb5. Clb5 is synthesized during S phase, when tRNA transcription peaks, and remains stable until mitosis when it is degraded by Cdc20. An earlier study reported that tRNA synthesis peaks during M phase [179]; however, in *S. cerevisiae*, the early phases of mitosis are known to overlap with S phase [183], and certain mitotic events occur simultaneously with S phase, which could also be the case for tRNA synthesis. Although it remains to be established exactly how the Clb5–Cdk1 complex is recruited to tRNA genes, it phosphorylates the TFIIIB component Bdp1 on N-terminal residues, thereby stimulating the interaction between TFIIIB and TFIIIC [180]. How this translates into increased RNAPIII activity is not yet clear, but one possible consequence may be an enhanced rate of facilitated recycling because RNAPIII residence time on chromatin is altered in mutants expressing non-phosphorylatable Bdp1 [180].

Interestingly, it has been shown that cell cycle-dependent expression of tRNA genes occurs at nuclear pores. Contact between tRNA genes and nuclear pores involves the nucleoporins Nup60 and Nup2, the Cohesin complex, and the tRNA exportin Los1 [179]. As already mentioned above, genes highly transcribed by RNAPII localize to nuclear pores in a manner dependent on Cdk1 activity [118]. It is, therefore, possible that Cdk1 promotes tRNA synthesis in at least two ways: By increasing the activity of the RNAPIII machinery as well as by localizing tRNA genes to a nuclear environment permissive for high transcriptional activity.

## 6. Conclusions

In addition to the well-described transcriptional programs that are controlled by Cdk1 through phosphorylation of specific transcription factors, it is clear that Cdk1 also directly controls basal transcription machineries. Direct regulation of various RNA polymerase complexes helps the cell maintain protein stoichiometry and cellular homeostasis. There are several notable differences in the timing and effect of cell-cycle CDKs on RNA polymerase machineries between yeast and vertebrates; in mammalian cells, the effect of Cdk1 has been studied mainly in M phase where it inhibits RNAPI, II, and III activities, whereas in yeast the effect of Cdk1 on RNAPII and RNAPIII occurs earlier in the cell cycle and has a positive effect on transcription. One possible explanation could be that in human cells the effect of early-acting cell-cycle CDKs (such as Cdk2, Cdk4, and Cdk6) on RNAPs is subtle or occurs only at a subset of genes. Consistent with this view, studies of transcriptional regulation by the HIV Tat protein have shown that Cdk2 directly phosphorylates RNAPII to promote transcription during G1/S phase, suggesting that CDKs can indeed have a positive effect on RNAPII in mammalian cells. It is also possible that the function of cell-cycle CDKs has diverged between yeast and vertebrates. After cell division, yeast daughter cells are substantially smaller than mother cells, and there may be a need specific to yeast cells for rapid protein synthesis to maintain protein stoichiometry and cell homeostasis during the rapid increase in daughter cell volume. More refined studies in vertebrate cells to specifically test the effect of cell-cycle CDKs on transcription using high-resolution methods will be required to clarify these differences.

## Figures and Tables

**Table 1:** CDKs and cyclins in *S. cerevisiae*.

CDK	Cyclin	Function
**Cdk1**		Essential CDK with similarity to mammalian Cdk1, main cell cycle regulator.
	Cln1, Cln2	G1 cyclins, regulate G1–S transition
	Cln3	G1 cyclin that is transcribed throughout the cell cycle, although protein levels peak in M phase. Regulates initial expression of Cln1 and Cln2
	Clb1,2	B-type cyclins involved in the transition from G2 to M phase
	Clb3,4	B-type cyclins mainly expressed in S–G2 phase and involved in spindle formation.
	Clb5, Clb6	S phase cyclins involved in DNA replication and transcription
**Pho85**	Clg1, Pcl1, Pcl2, Pcl5, Pcl6, Pcl7, Pcl8, Pcl9, Pcl10, Pho80	Non-essential CDK with similarity to mammalian Cdk5. Associates with ten different cyclins to regulate environmental and nutrient responses and to promote cell cycle progression.
**Bur1**	Bur2	CDK with similarity to mammalian Cdk9. Regulates transcriptional elongation through the phosphorylation of Ser2 of the CTD of RNAPII.
**Ctk1**	Ctk2	CDK with similarity to mammalian Cdk12 and Cdk13. Regulates transcriptional elongation through phosphorylation of Ser2 of the CTD of RNAPII.
**Kin28**	Ccl1	CDK with similarity to mammalian Cdk7. Involved in transcription initiation and mRNA capping through phosphorylation of Ser5 and Ser7 in the CTD of RNAPII.
**Ssn3**	Ssn8	CDK with similarity to human Cdk8. Part of the Mediator complex. Mainly involved in suppression of transcription.
